# Distinct neuroinflammatory patterns between cerebral microbleeds and microinfarcts in cerebral amyloid angiopathy

**DOI:** 10.1002/acn3.52226

**Published:** 2024-11-04

**Authors:** Laurent Puy, Romain Barus, Marco Pasi, Maud Pétrault, Vincent Deramecourt, Charlotte Cordonnier, Vincent Bérézowski

**Affiliations:** ^1^ Univ. Lille, Inserm, CHU Lille, U1172 ‐ LilNCog ‐ Lille Neuroscience & Cognition F‐59000 Lille France; ^2^ Department of Medical and Clinical Pharmacology, Centre of PharmacoVigilance and Pharmacoepidemiology Toulouse University Hospital Toulouse France; ^3^ Department of Neurology Tours Regional University Hospital, Hospital Trousseau Tours France; ^4^ Univ Lille, Lille Neuroscience & Cognition (Inserm UMRS1172) Development and Plasticity of the Neuroendocrine Brain Lille France; ^5^ Department of Neuropathology CHU Lille Lille France; ^6^ UArtois F‐62300 Lens France

## Abstract

In this neuropathological study, we investigated neuroinflammation surrounding recent and old cerebral microbleeds (CMBs) and cerebral microinfarcts (CMIs) in 18 cases of cerebral amyloid angiopathy (CAA). We used several serial stainings and immunolabellings to identify microvascular lesions, define their recent or old stage, and characterize neuroinflammatory response (scavenging activity and astrogliosis). We found that both CMBs and CMIs induce a neuroinflammatory response, which was more pronounced in old lesion than recent. Astrogliosis and scavenging activity were differentially prominent according to the ischemic/hemorrhagic nature of the lesion. Our findings provide insights into the pathophysiology of microvascular injuries in CAA.

## Introduction

Cerebral amyloid angiopathy (CAA) is a cerebral small vessel disease characterized by a widespread co‐occurrence of microvascular hemorrhagic (cerebral microbleeds, CMBs) and ischemic (cerebral microinfarcts, MIs) lesions.^1^ These lesions are now widely considered as markers of the severity of the disease. Recent advances confirmed that the majority of CMBs correspond to recent or old microhemorrhages with a progressive appearance of focal clusters of hemosiderin‐laden macrophages while old CMIs present tissue loss with cavitation.^2^ Nevertheless, beyond their neuropathological correlates, the histological consequences of CMBs and CMIs on adjacent brain tissue remain poorly understood, especially regarding the neuroinflammatory response (astrogliosis and scavenging activity). In this study, we aimed to characterize the neuroinflammatory changes surrounding both CMBs and CMIs in a postmortem analysis of patients with definite CAA. We hypothesized that both CMBs and CMIs exhibit neuroinflammation with specific patterns according to the nature (ischemic or hemorrhagic) and the stage (recent or old) of the lesion.

## Methods

### Human brain sampling

We led a postmortem study of 18 with a diagnosis of definite CAA patients. Cases were obtained from the Lille University Hospital brain bank (CRB/CIC1403 Biobank, BB‐0033‐00030, agreement DC‐2008‐642), which fulfills the criteria of the local laws and regulations on biological resources with donor consent, data protection, and ethical committee review. All patients had spontaneous ICH prior to death and were previously clinically followed up at the Lille University Hospital. Autopsies were performed within 12–36 hours after death, and hemispheres were fixed in formalin for 4–8 weeks. The sampling of brains involved relatively larger sections (average size: 6.5 cm) from different areas: frontal, parietal, and occipital lobes. Sections at the margin of ICH site were excluded to avoid the selection of acute CMBs and CMIs related to ICH.^3^ Neuropathologic diagnosis of definite CAA was confirmed by Red Congo staining and Amyloid β40 immunolabeling by one experienced neuropathologist (V.D).^4^

### Microvascular lesion assessment

All sampled tissue blocks were processed and embedded in paraffin, after which several 5‐μm thick serial sections were cut on a microtome. Sections were then processed for hematoxylin–eosin (H&E), Mariet scarlet blue (MSB), and Perl's coloration (for iron deposition). We used these stainings to assess the presence of microvascular lesions. Recent CMBs were characterized by the presence of abnormal small vessel wall and evidence for extravasation of red blood cells without iron deposits, whereas lesions with iron deposits were classified as old CMBs.^2^ Recent CMIs were defined by the presence of a circumscribed lesion characterized by a myelin loss (tissue pallor), vacuolization, and reduction in brain cells density in the vicinity of a small vessel (<500 μm in diameter). Old CMIs were characterized by tissue loss with cavitation and without iron deposition.^2^

### Neuroinflammatory marker assessment and quantification

Glial fibrillary acidic protein (GFAP) was used as molecular marker for activated astrocytes (astrogliosis). Furthermore, CD163 scavenger receptor expression was used as a surrogate marker of reactive amoeboid microglia and macrophages with scavenging activity. GFAP and CD163 immunolabelings were performed on a VENTANA BenchMark Immunohistochemistry automated staining machine. For each immunolabeling, a section was processed identically but with the omission of primary antibodies. A brightfield slide scanner (Zeiss AxioScan) at high resolution (20x) digitized whole tissue sections after staining. A staining surface ratio ([staining surface/whole section surface]*100) was used with ImageJ software to quantify the amount of immunolabeling expression within a 500 μm diameter from the epicenter of the microvascular lesions.

### Statistical analysis

Continuous, ordinal and categorical variables were expressed as the mean ± SD, the median [interquartile range] or the number (percentage), respectively. Univariate comparisons between recent and old CMBs were performed using Student *t*‐test for Gaussian continuous variables, Mann–Whitney U test for non‐Gaussian continuous and ordinal categorical variables, and chi‐square test for categorical variables. GraphPad Prism software was used to perform the statistical analysis, with *p*‐value considered significant when <0.05.

## Results

### Study population

The brains of 18 autopsy cases (all had an ICH, 7 males; median age at death = 78 [71–85] yo) with definite CAA were included in this study. On H&E, MSB and Perl's colorations we identified 71 microvascular lesions: 52 CMBs and 19 CMIs. Overall, 83% (*n* = 15/18) of the study population had at least one CMBs while 78% (*n* = 14/18) had at least one CMI. Among CMBs, 42 were recent and 10 were old. Among CMIs, 8 were recent and 11 were old.

### Neuroinflammation response between recent cerebral microbleeds and recent cerebral microinfarcts

**Figure 1:**
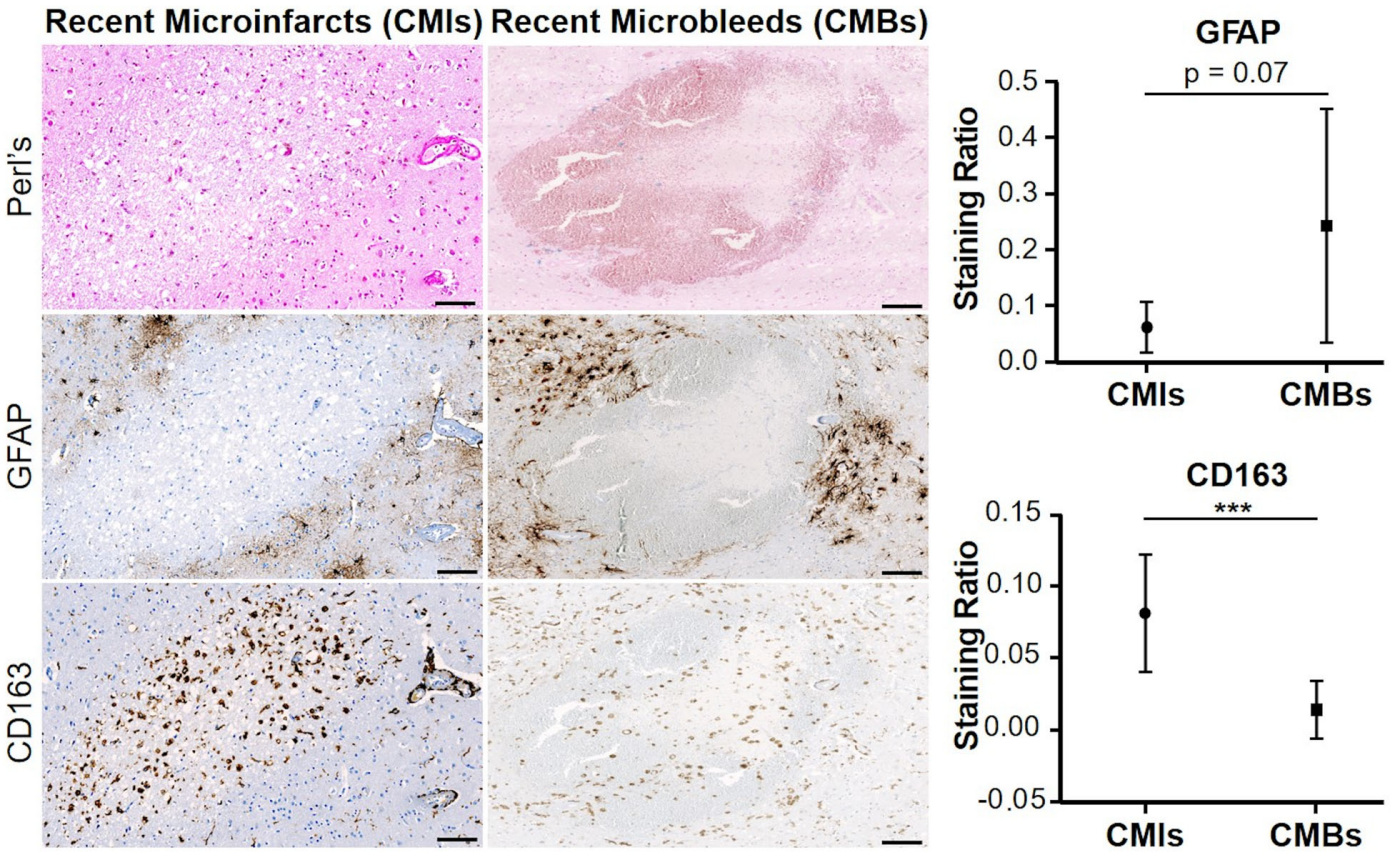
Neuroinflammatory pattern of recent cerebral microbleeds (CMBs) and recent microinfarcts (CMIs). The left panel shows a neuropathological illustration of a recent CMI and a recent CMB. From top to bottom, we show the Perl's staining (used to assess the recent or old stage), GFAP (activated astrocytes in brown), and CD163 (activated microglia and macrophages in brown) immunolabelings. The recent CMIs appeared as a circumscribed lesion characterized by a myelin loss (tissue pallor). The recent CMBs was characterized by an extravasation of fresh red blood cells with few iron deposition. These microvascular lesions presented a distinct inflammatory pattern according to their nature: recent CMIs exhibited more activated microglia and macrophages (cells with scavenging activity) than recent CMBs while activated astrocytes tended to be more abundant in recent CMBs than recent CMIs (see the right panel for statistical analysis). Scale bars = 100 μm. (**P < 0.001).

We observed neuroinflammatory changes in both recent CMBs and CMIs (Fig. 1). Astrocytes activation tended to be higher for recent CMBs (GFAP expression, *p* = 0.07) while recent CMIs exhibited more activated microglia with scavenging activity (CD163 expression, *p* < 0.001). Qualitative observations showed that recent CMIs were characterized by the presence of activated microglia with typical phenotypic changes in morphology (ameboid) as well as early brain tissue loss (see Fig. 1).

### Neuroinflammation response between old cerebral microbleeds and old cerebral microinfarcts

**Figure 2:**
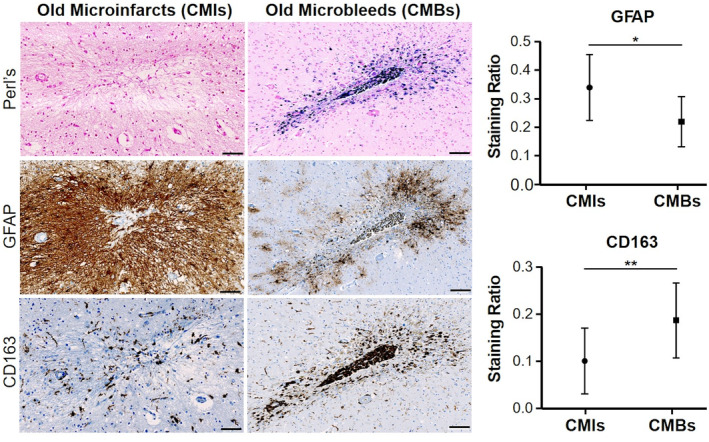
Neuroinflammatory pattern of old cerebral microbleeds (CMBs) and old cerebral microinfarcts (CMIs). The left panel shows a neuropathological illustration of an old CMI and an old CMB. From top to bottom, we show the Perl's staining (used to assess the recent or old stage), GFAP (activated astrocytes, in brown), and CD163 (activated microglia and macrophages, in brown) immunolabelings. Old CMIs were characterized by tissue loss, cavitation and gliosis and without iron deposition while old CMBs exhibit an abundant amount of iron deposition in the vicinity of an abnormal small vessel. Again, we observed a distinct inflammatory pattern according to the ischemic/hemorrhagic nature of the lesions: the astrogliosis reaction was higher in old CMIs than in old CMBs while the expression of cells with scavenging activity (activated microglia and macrophages) was more important in old CMBs (see the right panel for statistical analysis). Scale bars = 100 μm. ((*P < 0.005, **P < 0.001)

Both old CMBs and CMIs exhibited a higher expression of neuroinflammatory markers compared to recent lesions (*p* < 0.0001). Astrocytes activation was higher for old CMIs (GFAP expression, *p* = 0.035) while old CMBs exhibited more activated scavenging microglia (CD163 expression, *p* = 0.005) (Fig. 2). Qualitative observations showed a reactive pattern of microglia with an ameboid morphology and tissular loss within and around both CMIs and CMBs (Fig. 2).

## Discussion

Several findings emerged from this postmortem study regarding microvascular lesions in CAA: (i) CMBs and CMIs both induced a neuroinflammatory response that was more marked in old lesions than in recent ones; and (ii) this neuroinflammation exhibited distinct patterns and kinetics according to the hemorrhagic or ischemic nature of the lesion.

We found that the focal tissue response to both CMBs and CMIs is an intense and persistent neuroinflammatory reaction involving astrocytes (astrogliosis) and cells with scavenging activity (mostly microglia, and, in a lesser extent, recruited macrophages). This prolonged inflammatory response could cause subtle disruption of the local adjacent brain microenvironment homeostasis, and the accumulation of such events could contribute to the severity of the disease. Although it is difficult to state whether this inflammatory response is detrimental or beneficial, it is admitted that chronic neuroinflammatory state contributes to secondary tissue damages and delayed neuronal death.^5^ Hence, our findings concur with the literature refuting the presumed asymptomatic nature of microvascular lesions.^6^, ^7^, ^8^

Furthermore, we identified distinct inflammatory patterns according to the stage (recent versus old) and the nature (hemorrhagic versus ischemic) of the lesions suggesting two different pathophysiological processes. In CMIs, vessel occlusion induced a short‐term reaction with an early necrosis. This phenomenon was accompanied by an intense activation of microglia that rapidly switched from homeostatic to the activated amoeboid state to clear the debris cells through scavenging activity. Reactive astrogliosis peaks later to leave a gliosis scar within and around the infarcted area as observed by Yilmazer‐Hanke and collaborators.^9^ On the other hand, in CMBs, disruption induced a long‐term reaction involving more complex and delayed mechanisms: Red blood cells leakage induced iron accumulation and its toxicity will trigger and maintain a proinflammatory activity that will promote tissular damages and cell death.^10^ Overall, the time frame between vessel wall injury and cell death is probably longer for CMBs compared to CMIs. These distinctions between ischemic and hemorrhagic events may have therapeutic implications for future trial targeting CAA‐related small vessel disease progression since CMBs consequences could be more amenable to pharmacomodulation than CMIs. The link between microvascular injuries and inflammation might be more complex given that we studied the role of inflammation as a response to the formation of CMBs and CMIs, although growing body of evidence also suggests a causal role of inflammation in the disease process.^11^

We encountered a somewhat greater number of recent CMBs (78% contained intact erythrocytes) than anticipated. This discrepancy between the number of recent CMBs and old CMBs (ratio: 1 recent CMBs for 4 old CMBs) emphasized that not all CMBs become old in nature as previously suggested^12^ and that a spontaneous resolution, for a substantial proportion of recent CMBs, exists. Most recent CMBs were characterized by the presence of erythrocytes extravasation restricted to the perivascular spaces without parenchymal contamination, so we hypothesize that a blood leakage within the brain parenchyma is required for MBs to become chronic.

Our study has some limitations and strengths. The cross‐sectional nature of a histopathological study precludes the establishment of a precise chronology of events, so our “recent/old” comparison may lack precision. We used astrocytes and microglia activation as markers of inflammation but we acknowledge it does not cover the full spectrum of the neuroinflammatory response. We only investigated CAA cases, so whether our findings are similar in other small vessel disease such as arteriolosclerosis is unknown. In order to ensure a good reproducibility of our results, we used an immunohistochemistry automated staining machine and semi‐automated technique to quantify immunolabeling of the sections.

## Conclusion

Both CMBs and CMIs induce a neuroinflammatory response that increases from recent to old stage, and the profile of this response (astrogliosis versus scavenging activity) varied according to the nature of the lesion. These findings may have therapeutic implications for future trial targeting CAA‐related small vessel disease progression.

## Author Contributions

L.P., R.B., and V.D. contributed to the acquisition of the data and drafted the manuscript. L.P., R.B., M.P., V.D., V.B., and C.C. contributed to the interpretation of the data. All co‐authors reviewed the manuscript.

## Funding Information

This work was supported by the Fondation Recherche sur les Accidents Vasculaires Cérébraux (project FRAVC180713012), the Fondation pour la Recherche Médicale (FDM201806006375, fellowship L.P) and the Fondation I‐SITE ULNE (fellowship L.P).

## Conflict of Interest

R.B., M.P., M.P., V.D., and V.B.: None. L.P. declares the following type of interests: speaker fees (Novonordisk). C.C. declares the following type of interests: speaker fees (Bristol‐Myers Squibb); international RCT Steering committees for Biogen and Bayer.

## Data Availability

All data relevant to the study are included in the article or uploaded as supplementary information. Other data are available upon reasonable request.
